# The role of major duct excision and microdochectomy in the detection of breast carcinoma

**DOI:** 10.1186/1471-2407-6-164

**Published:** 2006-06-23

**Authors:** Mary F Dillon, Shah R Mohd Nazri, Shaaira Nasir, Enda W McDermott, Denis Evoy, Thomas B Crotty, Niall O'Higgins, Arnold DK Hill

**Affiliations:** 1 Department of Surgery, St Vincent's University Hospital, Elm Park, Dublin 4, Ireland; 2 Department of Pathology, St Vincent's University Hospital, Elm Park, Dublin 4, Ireland; 3 Department of Surgery, University College Dublin, Dublin 4, Ireland

## Abstract

**Background:**

The association of nipple discharge with breast carcinoma has resulted in numerous women undergoing exploratory surgery to exclude malignancy. The aim of this study was to determine whether pre-operative factors can identify those patients that are most at risk of carcinoma.

**Methods:**

All patients over a 14-year period (1991–2005) who had a microdochectomy or subareolar exploration for the evaluation of nipple discharge were assessed. Patient characteristics, pre-operative imaging and pathological findings were analysed.

**Results:**

Of the 211 patients included in this study, 116 patients had pathological (unilateral, uniductal serous or bloody) discharge. On excision, 6% (n = 7) of patients with pathological discharge and 2.4% (n = 2) of patients with non-pathological discharge were diagnosed with carcinoma. Overall, major duct excision resulted in the diagnosis of carcinoma in 4.3% (n = 9), ADH/LCIS in 4% (n = 8), papilloma in 39% (n = 83), and duct ectasia or non-specific benign disease in 53% (n = 111) of patients. In the patients determined to have malignancy, 44% (n = 4) were premenopausal. No patient with a non-bloody discharge in the total population analysed (28%; n = 59/211), or in the population with a pathological discharge (21%; n = 24/116) was found to have carcinoma upon excision.

**Conclusion:**

Microdochectomy or major duct excision performed for nipple discharge resulted in a low rate of malignancy on excision. Conservative management of non-bloody nipple discharge can be considered in patients with no other clinical or radiological signs of malignancy.

## Background

The association of pathological (unilateral, uniductal, spontaneous, serous or bloody) nipple discharge with breast carcinoma has resulted in numerous women undergoing exploratory surgery to rule out malignancy. The association of pathological nipple discharge with breast carcinoma is approximately 10–20% [1-7], but it may be considerably lower in patients with no obvious clinical or radiological evidence of breast carcinoma, who are undergoing major duct excision for diagnostic purposes [2,3,8]. This creates a considerable management challenge for surgeons as approximately 5% of patients present with discharge as a breast symptom [9-11]. A low rate of malignancy on excision may not justify routinely offering diagnostic surgery to all patients presenting with pathological nipple discharge, unless it is for the purpose of symptom relief. The ability to identify patients at low risk of malignancy, who may benefit from conservative management, would be valuable.

The purpose of this study was to determine the rate of malignancy following microdochectomy or subareolar exploration. Additionally, we sought to determine whether pre-operative risk factors identified those patients most at risk of malignancy. Finally, we evaluated the accuracy of routine preoperative tests in the diagnosis of significant pathology.

## Methods

This study was conducted in St Vincent's University Hospital, a tertiary referral centre for breast surgery. A surgical operating database, spanning a 14-year period, was queried for all patients who underwent a microdochectomy or subareolar exploration. This study excluded patients with a preoperative histological diagnosis that confirmed carcinoma and patients who did not undergo surgery for evaluation of nipple discharge. The study was conducted under the guidelines of the local ethics committee, and in accordance to the tenets of the Declaration of Helsinki.

All medical records of patients undergoing major duct excision surgery were reviewed. Data collected included patient age, menopausal status, nature and duration of discharge, associated clinical findings, family history of breast cancer, and findings from any imaging studies performed. In addition, diagnostic methods, operative procedure, and histopathological details were recorded for each case. Mammography was classified by radiologists using the BIRAD scale (BIRAD1& 2, benign; BIRAD 3, lesion of uncertain malignant potential; BIRAD 4, suspicious for malignancy; BIRAD 5, malignant). Ductograms were not performed preoperatively. Ultrasonography was introduced into clinical practice in the later years of the study.

The indication for subareolar exploration or microdochectomy in our institution was primarily pathological discharge. Pathological discharge was defined in this study as uniductal, unilateral, spontaneous, bloody or serous discharge. At the discretion of the treating consultant, patients who did not strictly fulfil the criteria for 'pathological' discharge or patients with persistent bothersome discharge, also underwent surgery. Surgery was performed using a circumareolar incision. In the microdochectomy procedure, a lacrimal probe was passed into the affected duct and the duct was excised. In the case of subareolar exploration, all the ducts behind the nipple were excised.

Statistical analysis was performed using SPSS (version 11, Chicago, IL) statistical software. Categorical variables were compared with Chi Squared Test, or Fishers' Exact Test (2-tailed), where appropriate. Continuous variables were analysed with the Mann Whitney U test.

## Results

### Patients

A total of 211 patients underwent a microdochectomy (n = 101) or subareolar exploration (n = 110) during the study period. Of these, 116 patients (55%) had pathological discharge and 86 patients (41%) had non-pathological discharge. There were 9 patients (4%) where incomplete data prevented categorisation of the nature of discharge [(spontaneous vs expressed (n = 7) and uniductal vs. multiductal (n = 5)] and where appropriate, these patients were excluded from the analysis. The median age of patients at the time of presentation was 49 years (range 22–78 years) for the total population, and 51 years (range 26–78 years) for patients with evidence of pathological discharge. Forty five percent of patients (n = 52) with pathological discharge were less than 50 years of age. Following excision, 4.3% (n = 9) of patients were diagnosed with malignancy, with a rate of 6% (n = 7) for patients with pathological discharge, and a rate of 2.4% (n = 2) for patients with non-pathological discharge.

### Discharge characteristics

The characteristics of the nipple discharge in the total population of patients who underwent an operation are shown in Table 1. Fifty nine patients (28%) were operated on for non-bloody discharge, 150 patients (71%) for bloody discharge, and 2 patients (1%) for serosanguinous discharge. Ninety five percent (n = 200) of patients had unilateral discharge, 79% (n = 158/201) had uniductal discharge and 79% (n = 159/202) had spontaneous discharge. Of those with pathological discharge, 21% (n = 24) had serous discharge.

### Preoperative examination

A clinical examination was performed on all patients. Nineteen patients (9%) had an associated palpable abnormality. While 2 of these 19 patients had malignancy, the lump in these 2 patients was not considered to be clinically suspicious. One of the 19 palpable abnormalities was considered suspicious for malignancy, but was found to be benign on excision. Seven patients with malignancy had no palpable abnormality. The sensitivity of clinical examination in this study was therefore determined to be 0%.

### Radiology

Mammographic details were available in 181 patients. 171 (94%) of these were benign (classified as BIRAD 1 or 2), 8 mammograms demonstrated definite lesions and 2 mammograms suggested ductal dilatation. No patient with carcinoma had findings on mammography that were suspicious for malignancy giving a sensitivity of mammography of 0% in this patient population. A mass or asymmetrical density was noted in 6 patients. The pathological finding on excision for these patients was papilloma (n = 2), and duct ectasia/non-specific findings (n = 4). Ductal dilatation was noted with 2 patients on mammography and papillomas were found on excision in both cases. Calcification (n = 1) requiring investigation (BIRAD 3 or above) was not associated with any specific findings, though 5 of the 9 patients with carcinoma had 'benign breast calcification' (BIRADS 1 or 2). Ultrasonography was performed on 33 patients. A correct cause of discharge was identified in only 3 of these patients (papilloma). Two patients with malignancy underwent ultrasound examination with non suspicious findings (duct ectasia n = 1), fibroadenoma n = 2).

### Excision pathology

Of the total group, 4.3% (n = 9) of patients were diagnosed with carcinoma on excision with a rate of 6% (n = 7) for pathological discharge, and a rate of 2.4% (n = 2) for non-pathological discharge. Two of the patients with malignancy were diagnosed with invasive carcinoma, and 7 with ductal carcinoma in situ (DCIS). The excision pathology findings for all patients are shown in Table 2. Those with serous discharge were found to have papilloma (n = 20), papilloma/atypical ductal hyperplasia (ADH) (n = 1), ductal ectasia (n = 5) or non-specific findings (n = 7). Patients with green, brown or milky discharge were diagnosed with ductal ectasia/benign breast findings in 83% (n = 15/18) of cases and papilloma/atypical papilloma in 17% (n = 3/18) of cases. The most common finding associated with bloody discharge was papilloma (41%; n = 61/150).

### Patients with malignancy

Data on patients who had a diagnosis of carcinoma following excision are provided in Table 3. Of the 9 patients, 5 underwent a microdochectomy procedure and 4 underwent a subareolar exploration. Of note, all 9 patients had bloody nipple discharge. Eighty nine percent (n = 8) had uniductal discharge and 78% (n = 7) had spontaneous discharge. There was no significant difference in those patients with malignancy versus those without, in respect to age, menopausal status, family history of breast carcinoma or the use of the oral contraceptive pill or hormone replacement therapy (Table 3). Forty four percent (n = 4) of patients with malignancy were pre menopausal.

### Specific lesions

Table 4 provides data on those patients who had normal or non-specific findings on excision and compares them to those with specific findings on excision. Of note, patients on hormone therapy (hormone replacement therapy or the oral contraceptive pill) were more likely to have non-specific findings (p = 0.024) on excision. A discharge colour other than bloody or serous was also associated with non-specific findings (p < 0.0001), whereas serous (p = 0.042) and uniductal disease (p = 0.006) were associated with specific findings.

### Recurrence of symptoms

Eighteen patients (9%) returned to clinic with a recurrence of discharge. The median time to recurrence of discharge was 7 months (range 0–5 years). In 12 patients (6%), the discharge returned within one year.

In addition, 3 patients were later diagnosed with malignancy at our clinic 2, 8 and 9 years following the initial resection. All three patients originally had bloody discharge, which was not present at the time of diagnosis of malignancy. However, two of the three patients presented at that time, with a lump near the nipple.

## Discussion

The association of pathological nipple discharge with malignancy, in a small proportion of patients, results in numerous women undergoing exploratory surgery to rule out evidence of serious pathology. Studies assessing pathological breast discharge report associated malignancy rates of up to 20% [1-7,12,13]. Those studies with high rates of malignancy usually include patients with obvious radiological malignancy, whose initial operative procedure was a diagnostic excision of a definite breast abnormality. These studies were often conducted prior to an era of widespread preoperative diagnosis with core biopsy. In contrast, in this study, we were concerned with the outcome of patients, who in the absence of an obvious cause of discharge, underwent major duct excision primarily as a diagnostic procedure. These patients present a particular challenge to surgeons and may have a different risk profile than those patients presenting with a more clinically and radiologically apparent breast carcinoma, and who also present with associated discharge.

In the present study of 211 patients, only 4.3% of patients were diagnosed with breast carcinoma following major duct excision. This includes a cohort of patients with pathological and non-pathological discharge, both of which were associated with a low risk of carcinoma (6% and 2.3% respectively). Overall, these results emphasise that the traditionally reported 10–20% rate of breast malignancy [4,6-8,14,15] associated with pathological discharge is not representative of those patients who present with pathological discharge alone. In a similar patient population to this study, King et al found only one case of malignancy out of 39 patients undergoing duct excisions [16], and in a study by Chaudary et al. [3] on patients undergoing microdochectomy, the carcinoma rate was 5.9%. Other studies have produced similar results [8,17]. Justification of diagnostic excision where there is such a low yield of malignancy becomes increasingly difficult, and emphasises the need for more discriminatory guidelines for this particular cohort of patients. In addition, it must be considered that excision of the ducts may not yield a diagnosis. In this study, a specific pathological cause of discharge was not identified in 25% of patients and in a further 25% 'duct ectasia/duct dilatation' was the only pathological finding. Our findings indicate that duct excision, used as a diagnostic test, has a sensitivity in the region of 50–75%. There is increasing evidence that a considerable number of pathological lesions are not located in close enough proximity to the nipple for a surgical duct excision to adequately locate them [5,18-20], which may further explain this low diagnostic yield.

The low rate of malignancy underpins the difficulty in identifying those patients most at risk of carcinoma. However, an important finding of this 14-year study was that none of the 59 patients who were operated on for a non-bloody discharge had malignancy on excision. The results of this study suggest that patients with a non-bloody discharge, who have no obvious clinical or radiological signs, are at low risk of malignancy. While there is certainly an association between non-bloody discharge and breast carcinoma [5,13,15,21], this association is more readily apparent in studies of patients with obvious breast abnormalities. In studies focusing on patients undergoing microdochectomies, whose indication for operation is for discharge alone, the clinical presentation appears to be different. A study by Chaudary et al. [3] on patients undergoing microdochectomy, with no associated lump, revealed that 5.9% were diagnosed with carcinoma. In that study, only two of the 16 patients who were diagnosed with carcinoma had serous discharge, but even these were hemoccult positive. In the study by Welch et al. [22], 14 of 16 patients diagnosed with carcinoma following microdochectomy had a bloody discharge. In other studies with this type of patient population, there is either no association [17], or a very low association [2,18,23] with carcinoma and non-bloody discharge. Occasionally, there are exceptions to these findings [5,13,24] such as in the study by Hou et al. [5], reporting on a Taiwanese population with a very high rate of carcinoma (20%) and an association with serous discharge, or the study by Sharma et al [13] demonstrating the association of carcinoma even with brown or purulent discharge. Essentially, however, it may be that small, sub-clinical cancers are more commonly associated with bloody discharge. In a patient population of this type, whose risk of carcinoma is typically low (approximately 5%), the evidence suggests that those with a non-bloody discharge are at particularly low risk, with a probable overall risk of malignancy of less than 1 or 2%. To put this in perspective, in the U.S. patients with mammographic lesions do not undergo (pre-operative) biopsy if their risk of malignancy, radiologically, is considered to be less than 2% [25] (BI-RADS 3). Therefore, it could be argued that this low risk sub-group with non-bloody discharge may also be monitored safely.

In this study, we also analysed those who had a specific cause for discharge identified following excision, and compared them to those with non-specific or normal findings. It was of interest, that patients on hormone therapy were found to be more at risk of non-specific findings on excision. Not surprisingly, discharge of a colour other than bloody or serous, was associated with non-specific disease, whereas serous discharge was associated with specific lesions. All the specific lesions in patients with serous discharge were papillomas including one case of papilloma and ADH. Currently, there is no consensus as to whether papillomas pose a significant risk of carcinoma or whether they should be removed [26-28]. Certainly, the finding that uniductal, as well as serous discharge, was associated with specific lesions supports the use of the term 'pathological' discharge in this category.

In the present study, the efficacy of various clinical parameters and tests were also analysed. While clinical history was the most useful indicator of risk with regard to the nature of discharge, age and menopausal status were of limited value. Forty four percent of patients diagnosed with carcinoma were under the age of 50, and were premenopausal. This contrasts with the findings of Lau et al. [23], who reported that 10 of 11 patients with cancer were postmenopausal and who subsequently recommended excision in all postmenopausal women with pathological discharge. Clinical examination was also found not to be discriminatory, most likely because patients with suspicious palpable abnormalities would have undergone a different procedure or would have had a preoperative histological diagnosis. All the patients with carcinoma in this study, even those with an associated palpable abnormality, were not considered suspicious on clinical examination.

Imaging in this study was also found to be unreliable. Only 9 mammograms demonstrated any abnormality and there were 8 false-negative mammograms. Ultrasound performed on 33 patients identified a cause of discharge in only 3 of the patients. The 2 patients with carcinoma who underwent an ultrasound examination were not diagnosed pre-operatively. The poor sensitivity of radiology, in this patient setting, is a reflection of higher pre-operative diagnosis rates. The more widespread use of core biopsies means that there are considerably less patients undergoing exploratory surgery for lesions visible on mammography. This change in practice is reflected by our high rate of false-negative mammography in diagnostic surgery and our very low overall malignancy rate. However, these results do suggest that routine ultrasonography of the retroareolar region, in the absence of mammographic abnormalities is of limited value.

Other potential pre-operative tests such as ductography (galactography) or ductoscopy are not performed in our unit. Ductography was introduced over 60 years ago [29], but has not become standard practice in many parts of the world [6,30,31]. It has not proved to be very effective at discriminating between benign and malignant lesions, and a negative test does not exclude malignancy [1]. Likewise, ductoscopy is also limited by its low specificity [18]. Despite these disadvantages, ductoscopy and ductography appear to play an important role in guiding a more conservative excision or in localising lesions at some distance from the nipple [18,19]. Several studies have indicated that lesions are frequently found at some distance from the nipple orifice [5,18-20,32]. More disturbingly, Hou et al. [5] reported that 70% of patients with carcinoma presenting with discharge had their lesions located > 2 cm from the nipple [5].

These studies imply the need for localisation techniques. Localisation of lesions appears to be most effective if done at the time of operation. In a study by Cabioglu et al [7] most patients had preoperative ductography performed. However it was patients who underwent a ductography guided operation or that had a localising study at the time of operation, who where significantly more likely to have a specific underlying lesion identified, as compared to patients who underwent a central duct excision alone. Similarly, Van Zee et al. [33] demonstrated the value of immediate preoperative ductography in identifying specific lesions, though a more recent study suggested that localisation through means of ductoscopy guided ductal excision may not be as effective [19]. Our study had a high rate of non-specific or benign findings on excision, indicating that a surgical procedure alone may not be adequate as a diagnostic tool. Increasing interest into more accurate techniques to identify lesions, including the use of thinner more flexible fiberoptics ductoscopy [34] may enhance the value of surgical excision.

## Conclusion

In this study, patients undergoing major duct excision for evaluation of pathological nipple discharge had a low rate of malignancy on excision. This low rate of malignancy, coupled with the finding that no patient operated on in the 14-year period for non-bloody discharge had malignancy on excision, suggests that a conservative approach may be considered in patients with non-bloody discharge. The results of our study also suggest that patient age is not helpful in identifying those at risk of malignancy following major duct excision, and that major duct excision has limitations as a diagnostic tool.

## Competing interests

The author(s) declare that they have no competing interests.

## Authors' contributions

MD- study conception

MD, SN, SMN- study design

SN, SMN-data acquisition

MD- data analysis

MD- drafting of manuscript

AH, EMD, TC, DE, NOH-critical revision of manuscript

## Source of funding

There was no source of funding

## Pre-publication history

The pre-publication history for this paper can be accessed here:



## Figures and Tables

**Table 1:** Nature of discharge

	Total group [n = 211(%)]	Non-pathological discharge* [n = 86 (%)]	Pathological discharge* [n = 116 (%)]
Bloody	150 (71%)	51 (60%)	91 (78%)
Serosanguinous	2 (1%)	2 (2%)	
			
Non-bloody	59 (28%)	33 (38%)	24 (21%)
*Serous*	*33 (16%)*	*7 (8%)*	*24 (21%)*
*Green*	*13 (6%)*	*13 (15%)*	
*Brown*	*5 (2%)*	*5 (6%)*	
*Other*	*8 (4%)*	*8 (9%)*	
			
Unilateral	200 (95%)	75 (87%)	116 (100%)
Bilateral	11 (5%)	11 (13%)	
			
Uniductal	158 (79%)†	38 (47%)†	116 (100%)
Multiductal	43 (21%)†	43 (53%)†	
Unspecified	10	5	
			
Spontaneous	159 (79%)†	41 (49%)†	116 (100%)
Expressed	43 (21%)†	43 (51%)†	
Unspecified	9	2	

*Nine patients could not be classified as either pathological or non-pathological due to unavailable data

† Percentages expressed of available data

**Table 2:** Pathological findings on excision

	Total group [n = 211]	Non pathological discharge* [n = 86]	Pathological discharge* [n = 116]	Bloody discharge [n = 152]	Non bloody discharge [n = 59]
Papilloma	81 (38%)	26 (30%)	52 (45%)	59 (39%)	22 (37%)
Papilloma and ADH/atypical papilloma	5 (2%)	3 (3%)	2 (2%)	2 (1%)	3 (5%)
ADH/LCIS†	5 (2%)	2 (2%)	3 (3%)	5 (3%)	0 (0%)
Carcinoma	9 (4.3%)	2 (2%)	7 (6%)	9 (6%)	0 (0%)
Duct ectasia	51 (24%)	24 (28%)	25 (22%)	34 (22%)	17 (29%)
Benign/non-specific findings	60 (28%)	29 (34%)	27 (23%)	43 (28%)	17 (29%)

Abbreviations: ADH = atypical ductal hyperplasia; LCIS = lobular carcinoma in situ

† LCIS was of the 'classical' variant

*Nine patients could not be classified as either pathological or non-pathological discharge

**Table 3:** Patient characteristics of those with malignant histology versus those without.

	Patients with Malignant Lesions [n = 9]	Patients with non-malignant lesions [n = 202]	P-value
Median age (range)	54 (45–75)	49 (22–78)	0.32
Family history	3 (33%)	33/199† (17%)	0.19
Hormone replacement therapy/oral contraceptive use	1 (11%)	55/188† (29%)	0.45
Premenopausal	4 (44%)	104 (51%)†	0.7
Associated lump	2 (22%)	17 (8%)	0.19
			
*Discharge colour*			
Serous	0 (0%)	33 (16%)	0.36
Bloody	9 (100%)	141 (70%)	0.06
Serosanguinous	0 (0%)	2 (1%)	1
Other	0 (0%)	26 (13%)	0.6
Brown	0 (0%)	5 (2%)	1
Milky/pus	0 (0%)	8 (4%)	1
Green	0 (0%)	13 (6%)	1
			
Unilateral	9 (100%)	191 (95%)	1
Bilateral	0 (0%)	11 (5%)	1
			
Uniductal	8 (89%)	150 (78%)†	0.45
Multiductal	1 (11%)	42 (22%)	0.45
Unspecified		10	
			
Spontaneous	7 (78%)	152 (79%)	1
Expressed	2 (22%)	41 (21%)	1
Unspecified		9	
			
Radiology			
*Mammography*	n = 9	n = 172	
Normal/benign findings (BIRADS 1 or 2)	9 (100%)	164 (95%)	1
Abnormal findings (BIRADS 3–5)	0 (0%)	8 (5%)	1
			
*Ultrasound*	n = 2	n = 31	
			
Normal findings (BIRADS 1–2)	1 (50%)	26 (84%)	0.3
Abnormal findings (BIRADS 3–5)	1 (50%)*	5 (16%)	0.3

†Percentages are expressed of available data

* 'Fibroadenoma'

**Table 4:** Patient characteristics in those with specific lesions on excision versus those with non-specific findings or duct ectasia.

	Non-specifc findings/Duct ectasia [n = 111]	Specific Lesion [n = 100]	P-value
Median age (range)	49 (22–78)	49 (26–75)	0.7
Family history	17/108 (16%)	20/97 (21%)	0.37
Hormone replacement therapy/oral contraceptive use	36/101 (36%)	20/95 (21%)	0.024
Premenopausal	56(50%)	52 (52%)†	0.86
Associated lump	10/109 (9%)	9/99 (9%)	0.98
			
*Discharge colour*			
Serous	12 (11%)	21 (21%)	0.042
Bloody	77 (69%)	75 (75%)	0.36
Other	22 (20%)	4 (4%)	<0.001
Brown	4 (4%)	1 (1%)	0.37
Milky/pus	7 (6%)	1 (1%)	0.068
Green	11 (10%)	2 (2%)	0.017
			
Unilateral	103 (93%)	97 (97%)	0.17
Bilateral	8 (7%)	3 (3%)	0.17
			
Uniductal	73 (66%)	85 (85%)	0.006
Multiductal	30 (27%)	13 (13%)	0.006
Unspecified	8 (7%)	2 (2%)	
			
Spontaneous	85 (77%)	74 (74%)	0.59
Expressed	21(19%)	22 (22%)	0.59
Unspecified	5 (5%)	4 (4%)	
			
Radiology			
*Mammography*	n = 94	n = 87	
Normal/benign findings (BIRADS 1 or 2)	88 (94%)	85 (98%)	0.28
Abnormal findings (BIRADS 3–5)	6 (6%)	2 (2%)	0.28
			
*Ultrasound*	n = 16	n = 17	
Normal findings (BIRADS 1–2)	15 (94%)	12 (71%)	0.18
Abnormal findings (BIRADS 3–5)	1 (6%)	5 (29%)	0.18
